# RNA virus building blocks—miRNAs not included

**DOI:** 10.1371/journal.ppat.1006963

**Published:** 2018-05-31

**Authors:** Lauren C. Aguado, Benjamin tenOever

**Affiliations:** 1 Laboratory of Virology and Infectious Disease, Rockefeller University, New York City, New York, United States of America; 2 Department of Microbiology, Icahn School of Medicine at Mount Sinai, New York City, New York, United States of America; University of Florida, UNITED STATES

## Introduction

In most eukaryotic organisms, small noncoding RNAs called microRNAs (miRNAs) post-transcriptionally silence mRNAs to fine-tune protein expression. This mechanism, having likely evolved from a duplication of a small-RNA–mediated antiviral defense system, now orchestrates various developmental processes and is essential for maintaining cellular homeostasis [1]. In contrast to antiviral RNA interference (RNAi), miRNAs derive from host-encoded RNA hairpins, and their maturation requires sequential nuclear and cytoplasmic processing events using two RNase III nucleases: Drosha and Dicer, respectively [2]. The miRNA is then loaded into the Ago-based RNA-induced silencing complex (RISC) and engages a target mRNA in a sequence-specific manner [2]. The impact a miRNA has on its cognate target is determined by the extent of complementarity. Typically, binding of the miRNA to the target mRNA is not contiguous, leading to only moderate changes in RNA and protein levels, yet it can dramatically influence cellular processes with prolonged activity. In contrast, when the small RNA engages its target with perfect complementarity, the mRNA is cleaved, leading to complete ablation of the message.

Given the potential influence miRNAs can exert on their host, it is not surprising that many DNA viruses have been found to encode their own variants [3]. Curiously, though, utilization of miRNAs appears to be completely absent from RNA viruses that lack a DNA intermediate, suggesting that there are inherent constraints responsible for this discrepancy [4]. Here, we examine the originally hypothesized restrictions on miRNA exclusion, present evidence addressing these ideas, and propose an alternative hypothesis to explain the lack of their utilization amongst RNA viruses.

## Theoretical constraints on miRNA production from RNA viruses

**Fig 1:**
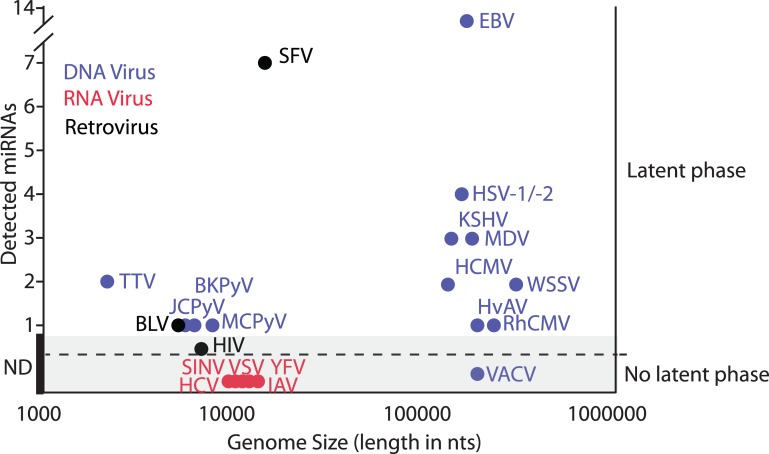
Depiction of viruses for which small-RNA populations have been sequenced in an attempt to identify putative miRNAs. Viruses are plotted based on confirmed miRNA numbers (left axis) versus both their life cycle (latent or nonlatent, right axis) and their overall genomic size (*x*-axis). ND denotes no miRNAs detected. DNA viruses (blue) include the following: BKPyV, JCPyV, MCPyV, VACV, RhCMV, HvAV, WSSV, HCMV, MDV, KSHV, HSV-1/-2, and EBV. Retroviruses (black) include the following: HIV, BLV, and SFV. RNA viruses (red) include the following: YFV, SINV, HCV, VSV, and IAV. BKPyV, BK polyomavirus; BLV, bovine leukemia virus; EBV, Epstein–Barr virus; HCMV, human cytomegalovirus; HCV, hepatitis C virus; HIV, human immunodeficiency virus; HSV-1/-2, herpes simplex virus-1/-2; HvAV, *Heliothis virescens* ascovirus; IAV, influenza A virus; JCPyV, JC polyomavirus; KSHV, Kaposi sarcoma-associated herpes virus; MCPyV, Merkel cell polyomavirus; MDV, Marek disease virus; miRNA, micro-RNA; RhCMV, Rhesus cytomegalovirus; SFV, simian foamy virus; SINV, Sindbis virus; VACV, vaccinia virus; VSV, vesicular stomatitis virus; WSSV, white spot syndrome virus; YFV; yellow fever virus.

Viruses are obligate intracellular parasites that require the resources of the cell to amplify and spread. In many cases, the process of virus replication is optimized through the manipulation of the cell to ensure their defenses are minimized and the resources required for viral replication are maximized. These cellular alterations are most commonly orchestrated by virus-encoded proteins but can also be mediated through miRNAs. This latter strategy is best exemplified by the Herpesviridae family, which has been found to encode anywhere from one to fourteen different miRNAs [3]. Efforts to define the function of these known virus-derived miRNAs have accredited them with two major functions. The first includes regulating levels of viral products directly and thereby orchestrating the transition across different stages of their replication cycle. The second includes acting as mimics for endogenous cellular miRNAs and modulating host transcripts, ultimately resulting in an environment more amenable to replication and/or latency [5, 6]. These utilizations of miRNAs appear to be ubiquitous amongst many nuclear viruses that establish latent infections within a target cell (Fig 1). While the majority of miRNA-utilizing viruses have large DNA genomes, a number of small retroviruses are included in this category as well [3].

**Fig 2:**
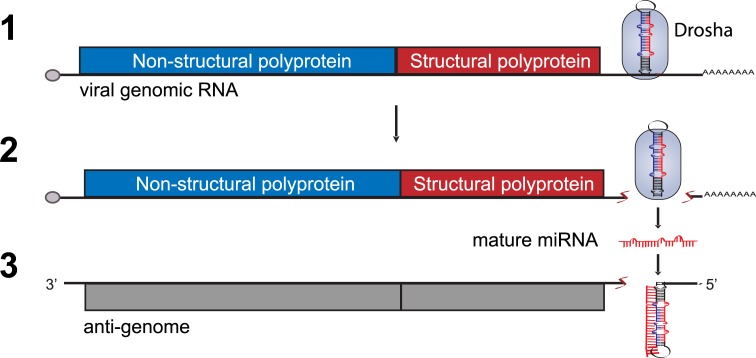
Depiction of the theoretical constraints to explain the absence of RNA-virus–encoded miRNAs. **(1)** Drosha, the RNase III nuclease required for the initial processing step in miRNA biogenesis, was thought to be exclusively nuclear. However, during infection, Drosha can translocate, allowing access to the cytoplasmic viral genomic material. **(2)** Drosha engagement with and subsequent processing of RNA-virus–encoded hairpins leads to destruction of the genome. **(3)** After further processing, the resulting mature miRNA would be perfectly complementary to the viral antigenome. RISC engagement of this complementary region within the antigenome would lead to slicing and thus rapid destruction of the target. miRNA, microRNA; RISC, RNA-induced silencing complex.

In contrast, acute cytoplasmic viruses, regardless of genome composition or size, do not appear to usurp this aspect of host cell biology (Fig 1). The lack of evidence for RNA-virus–mediated miRNA production was previously thought to be due to any one of three reasons [4]. These include the following: 1) the necessary machinery for biogenesis would be inaccessible in the cytoplasm where most RNA viruses replicate; 2) excision of the pre-miRNA from the primary transcript would result in destruction of many RNA-based viral genomes; 3) the mature miRNA may result in self-cleavage of the virus’s own antigenome (Fig 2). Below, we discuss recent discoveries that have assessed these theoretical constraints and found that none of them adequately explain why cytoplasmic RNA viruses do not appear to utilize their own miRNAs. As an alternative, we offer a new hypothesis to explain the absence of RNA-virus–encoded miRNAs.

## Can they even reach the tools?

RNA-virus replication predominantly occurs in the cytoplasm. As such, generation of a virus-derived miRNA would demand production of a Dicer-compatible hairpin. While this substrate could be generated in a noncanonical manner, it is usually the product of Drosha cleavage in the nucleus. For this reason, the first efforts to address why RNA viruses do not use miRNAs focused on the engineering of influenza A virus, which replicates in the presence of Drosha. Surprisingly, this pri-miRNA–containing recombinant virus permitted generation of a functional miRNA without any evidence of attenuation [7, 8]. Subsequent to this, multiple groups built upon this work to show that even cytoplasmic RNA viruses could be engineered to demonstrate the same phenotype [9, 10]. Shapiro and colleagues and Rouha and colleagues were the first to independently describe recombinant cytoplasmic viruses that could successfully produce miRNAs from a pri-miRNA substrate, establishing that access to Drosha is not a limiting factor [9, 10]. Since these initial discoveries, many other cytoplasmic viruses have been demonstrated to support miRNA production; these viruses include vesicular stomatitis virus (VSV), vaccinia virus (VacV), Semliki Forest virus (SFV), turnip crinkle virus (TCV), and Sendai virus (SeV) [11–13]. In each case, miRNA production is dependent on the translocation of Drosha following virus infection, which can be observed in diverse eukaryotic species, including mammals, zebrafish, arthropods, and plants, raising many questions regarding the evolutionary origins of this process [13, 14].

## Cutting the genome seems like a bad idea

Despite translocation of the necessary miRNA processing machinery, it would stand to reason that the excision of a hairpin embedded in a positive-stranded RNA virus would be deleterious. Indeed, hairpin-mediated loss of viral titers has been observed with recombinant lentiviruses and has been found to demand the use of DNA-dependent RNA polymerase III in other retroviruses [5, 15, 16]. Given this, it would seem that generating a Drosha substrate within a single polycistronic mRNA, a common genomic architecture for positive-stranded RNA viruses, would significantly constrain utilization of miRNAs. However, even attenuation that could occur as a result of genomic processing of positive-stranded RNA viruses does not appear to be an obvious constraint, as miRNA production can be achieved in such viruses without any obvious loss of fitness [9, 10]. While engineering RNA viruses can result in attenuation, it appears that this is largely context-specific. For example, alphaviruses encoding pri-miRNAs as either genomic or subgenomic components, despite producing copious amounts of virus-derived miRNAs, do not universally show attenuation in vitro or in vivo [17]. As cleavage of hairpin-containing RNA clearly occurs, lack of observed attenuation is likely due to the high production of template. Based on next-generation sequencing, we estimate that only a small percentage (less than 10%) of viral pri-miRNAs are processed by Drosha. This, in turn, appears to have little to no impact on virus replication, while still generating thousands of copies of a given miRNA per infected cell [14]. Interestingly, this phenotype appears to be somewhat specific to the location of the hairpin within the viral genome. When these hairpins are found within the immediate 5′ region of positive-sense RNA viruses, replication can be significantly dampened through engagement by the host small-RNA machinery [13]. Taken together, these observations suggest that while pri-miRNAs are not inherently detrimental to an RNA virus, there are some constraints on where they could be placed based on the virus’s genomic organization.

## When complements are not kind

The last theoretical constraint that might explain the lack of miRNA production from cytoplasmic RNA viruses derives from the concept of self-targeting. Because RNA virus-produced miRNAs would be perfectly complementary to the antigenome, one would imagine that this could cause dramatic attenuation. However, while small-RNA access could be hindered in both negative-stranded RNA viruses, through the formation of ribonucleoprotein complexes, or dsRNA viruses, which are already perfectly paired RNA duplexes, positive-stranded viruses should, in theory, be accessible for base-pairing with the miRNA and thus susceptible to self-targeting. However, here again, when one designs a positive-stranded RNA virus to encode a miRNA and then monitors antigenome production directly, there is no evidence of self-cleavage, likely owing to virus compartmentalization during replication. Alternatively, targeting of the antigenome could be prevented by engagement of the RNA-dependent RNA polymerase (RdRp) and parallel production of new genomes. In either instance, alphaviruses, rhabdoviruses, orthomyxoviruses, and carmoviruses have all been shown to successfully generate miRNAs without any obvious loss to viral fitness [7, 9–11, 13].

## So why don’t cytoplasmic RNA viruses make miRNAs?

Given the observed experimental results, it remains a wonder as to why RNA viruses do not co-opt cellular miRNA machinery as a means of enhancing their replication. One possible explanation for the lack of miRNA utilization among acute RNA viruses could be due to kinetics. As fine-tuners of protein levels, the impact of miRNA-mediated post-transcriptional silencing occurs over the course of days, not hours [18]. Therefore, it stands to reason that the only viruses generating miRNAs are those that persist within an infected cell, and these tend to be DNA viruses or RNA viruses with DNA phases (Fig 1). However, the kinetics of miRNA-mediated repression is the result of limited complementarity to a given mRNA. When miRNAs engage their target with perfect complementarity, cleavage of the RNA can occur within the time frame of an acute virus infection. Even in this scenario, the virus would only benefit from silencing if levels of the corresponding protein were low at the time of RNA cleavage. Interestingly, these dynamics could be observed when artificial miRNA libraries were inserted into viruses and used to select for increased fitness. In each case, target selection corresponded to proteins that were not expressed at baseline [17, 19]. These findings suggest that the pool of targets to enhance virus replication would be severely limited and thus may explain, in part, why this strategy is not utilized.

Limited host targets aside, the most compelling explanation for the absence of RNA virus-encoded miRNAs is that this strategy is simply not as advantageous as a protein with regards to cellular manipulation. By nature, the miRNA would need to be perfectly complementary in order for it to be rapidly beneficial, and this would likely render this strategy target-specific. In contrast, a viral protein can be multifunctional and engage many factors at the protein level. While it could be argued that the virus might circumvent this shortcoming by producing multiple miRNAs, a tandem array of RNA hairpins has been found to come at a fitness cost [20].

## Conclusion

Given the observations and current understanding of miRNA biology, it would seem safe to conclude that the selective utilization of virus-encoded miRNAs by persistent viruses is due to the limited advantages mRNA targeting can instill during an acute infection. While it remains possible that an acute RNA virus could benefit from the modulation of a single host mRNA, this is likely not observed because such an activity can be achieved more broadly and efficiently through use of a protein-based antagonist. Moreover, although initial efforts have failed to identify any obvious fitness cost from the inclusion of a single miRNA, more in-depth studies under greater selective pressures may reveal that the inclusion of a hairpin does consistently impact virus replication in a negative way. While future work may still one day identify an acute RNA virus that generates a bona fide miRNA, based on our current knowledge, it would appear that such a strategy confers a gain that does not impart any evolutionary benefit.
